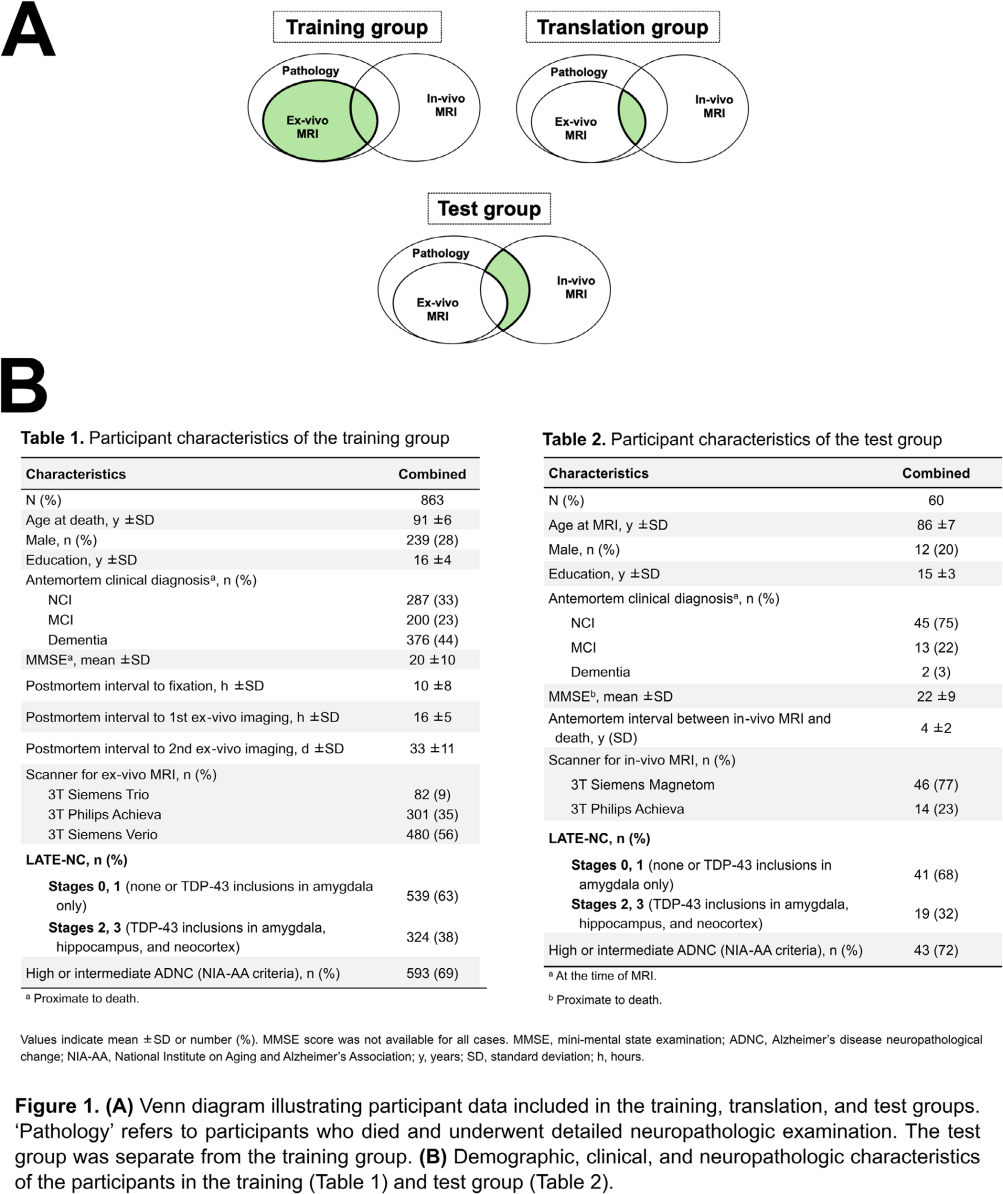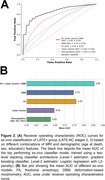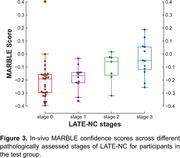# Predicting Limbic‐predominant Age‐related TDP‐43 Encephalopathy (LATE) based on In‐vivo MRI Features

**DOI:** 10.1002/alz70862_109757

**Published:** 2025-12-23

**Authors:** Mahir Tazwar, Arnold M Evia, Abdur Raquib Ridwan, David A. A. Bennett, Julie A Schneider, Konstantinos Arfanakis

**Affiliations:** ^1^ Illinois Institute of Technology, Chicago, IL USA; ^2^ Rush University Medical Center, Chicago, IL USA

## Abstract

**Background:**

Limbic‐predominant age‐related TDP‐43 encephalopathy neuropathologic change (LATE‐NC) is a common pathologic finding in aged brain, however, definitive diagnosis of this disease is only possible at autopsy. This work aimed to develop a marker of LATE‐NC based on in‐vivo MRI features from a large group of community‐based older adults.

**Method:**

This study included ex‐vivo MRI, in‐vivo MRI, and pathology data from four longitudinal clinicopathological cohort studies of aging conducted at the Rush Alzheimer’s Disease Center (ROS, Rush MAP, MARS, LATC). LATE‐NC was evaluated based on pathologic TDP‐43 inclusions in 8 brain regions and categorized into 4 stages (Figure 1). MRI data was processed to obtain fractional anisotropy (FA), deformation‐based morphometry (DBM) measurements, and lobar volumes.

To develop a marker of LATE‐NC, we first trained a classifier to distinguish between advanced (stages 2‐3) and early LATE‐NC stages (stages 0‐1) based on ex‐vivo MRI features (*N* = 863). Our classifier used a two‐level stacking model for training and cross‐validation, where level‐1 estimators generated risk scores based on single‐modality features, and level‐2 estimator (logistic regression) provided the final LATE‐NC prediction score based on the combined risk scores. We then translated the classifier to in‐vivo and validated it on a separate set of participants (*N* = 60) with in‐vivo MRI and pathology data. The entire pipeline was packaged into an automated software container named MARBLE (MARker of Brain LatE).

**Result:**

In the training group, the ex‐vivo classifier demonstrated excellent performance, achieving an average AUC of 0.85±0.05 (sensitivity=78%, specificity=76%, balanced accuracy=77%) based on FA, DBM, and lobar volume features (Figure 2). In‐vivo validation of MARBLE scores yielded an overall AUC of 0.76 in the test group. Additionally, ordinary least‐squares regression revealed higher MARBLE scores with greater LATE‐NC stages (*p* <0.001), controlling for antemortem interval (AMI) and scanners (Figure 3).

**Conclusion:**

This study developed MARBLE, a novel, automated, in‐vivo marker of LATE‐NC based on MRI features. MARBLE was trained on ex‐vivo MRI and pathology data from a large number of community‐based older adults and showed decent performance in‐vivo (AUC=0.76). While further validation is needed in independent cohorts, MARBLE has the potential to significantly contribute towards in‐vivo diagnosis, monitoring, prevention, and treatment of LATE‐NC.